# A review of music performance anxiety in the lens of stress models

**DOI:** 10.3389/fpsyg.2025.1576391

**Published:** 2025-04-16

**Authors:** A. J. Twitchell, A-A. Journault, K. K. Singh, J. P. Jamieson

**Affiliations:** ^1^Independent Researcher, Florianopolis, Brazil; ^2^Department of Psychology, University of Rochester, Rochester, NY, United States

**Keywords:** music performance anxiety, biopsychosocial model, stress, stress optimization, review

## Abstract

Music performance anxiety (MPA) is a widespread challenge among musicians, often impairing performance quality and well-being. While traditionally conceptualized as a debilitating condition, recent research suggests that MPA can have both facilitative and detrimental effects, depending on how it is appraised and regulated. This paper reviews theoretical stress models relevant to MPA, emphasizing frameworks such as stress optimization and synergistic mindsets that help reframe anxiety as a potential resource. By integrating insights from affective science and stress research, this review highlights the need for a more nuanced approach to MPA—one that moves beyond symptom reduction to foster adaptive responses that enhance musical performance. Future research should continue exploring personalized and flexible interventions that equip musicians with the tools to navigate evaluative pressure effectively.

## 1 Introduction

Performing in evaluative settings is inherently stressful. For musicians performing to an audience, the stressfulness of the situation is further increased by additional demands to the task of playing the instrument, such as evaluative pressure from the self and others and consequences for one’s reputation or vocational prospects. In many cases, the burden of demands leads to anxiety and harms the musician’s performance, a phenomenon described as debilitative music performance anxiety (MPA). The current paper reviews research from stress, affective science, and social psychology to provide an integrated model for understanding MPA and leverages recent advances in affective science to inform approaches for optimizing outcomes in these contexts.

MPA has been defined in many ways, ranging from its equivalence to stage fright (Ray, 2009; Steptoe, 2001) to a graded phenomenon that spans from light apprehension to full-blown panic (Maciente, 2016; Wilson, 2002). Still, other conceptualizations refer to MPA as a cluster of intense symptoms resembling a pathological disorder, which impairs performance and requires treatment (Brugués, 2019). Although definitional discrepancies exist, Kenny (2009) definition has become increasingly relied upon describing MPA as an “experience of marked and persistent anxious apprehension related to musical performance” (Kenny, 2009, p. 433), characterized by a combination of affective, cognitive, somatic, and behavioral symptoms. It may harm the quality of the performance but does not necessarily do so.

The idea that performance anxiety can be both adaptive and maladaptive is supported by some empirical research (Connolly and Williamon, 2004; Gültepe and Coskun, 2016; Hanin, 2010; Kenny and Ackermann, 2009; Mac Afee and Comeau, 2020; Osborne et al., 2014). In seeking to identify elements that contribute to facilitative or debilitative MPA, a common assumption is that both low and high levels of anxiety are debilitative, while medium-level anxiety is facilitative (Paliaukiene et al., 2018; Sinico da Cunha and Winter, 2013; Steptoe and Fidler, 1987; Valentine, 2004). Despite the existence of two-sided views of MPA, many studies have focused on the debilitating symptoms of MPA, consistently finding a high prevalence among musicians (Casanova et al., 2018; Kenny et al., 2016; Miller and Chesky, 2004; Wesner et al., 1990; Roland, 1994). However, beyond examining strategies to mitigate the debilitating effects of anxiety on musical performance, there is a need to explore how MPA may also be beneficial (Huang and Yu, 2022; Mac Afee and Comeau, 2020; Jamieson et al., 2018a,b; Crum et al., 2017; Osborne et al., 2014). Indeed, while a few notable studies situated MPA within a unified and coherent explanatory model to inform future studies and interventions (e.g., LeBlanc, 2021; Kenny, 2004; Wilson, 2002; Papageorgi et al., 2007), these theories rely on outdated research. The field of MPA would benefit from the advances made in affective science over the last 20 years to offer more practical insights and guidance for musicians.

## 2 Stress theories

### 2.1 Biopsychosocial model of challenge and threat

The complex and differential impacts of MPA on musicians and performance outcomes align with modern theories of stress, and in particular, biopsychosocial (BPS) models, which have become dominant in the medical field. BPS models emphasize the multifaceted nature of stress responses, driven by biological, psychological, and contextual interactions. Thus, they provide a valuable framework for understanding both maladaptive and adaptive MPA generation, offering insights into how to downregulate the former and upregulate the latter. While much of the existing research using BPS models to understand performance outcomes has predominantly focused on sports or academic performance, rather than music, we theorize that the processes they describe may offer valuable insights for advancing our understanding of MPA.

**Figure 1:**
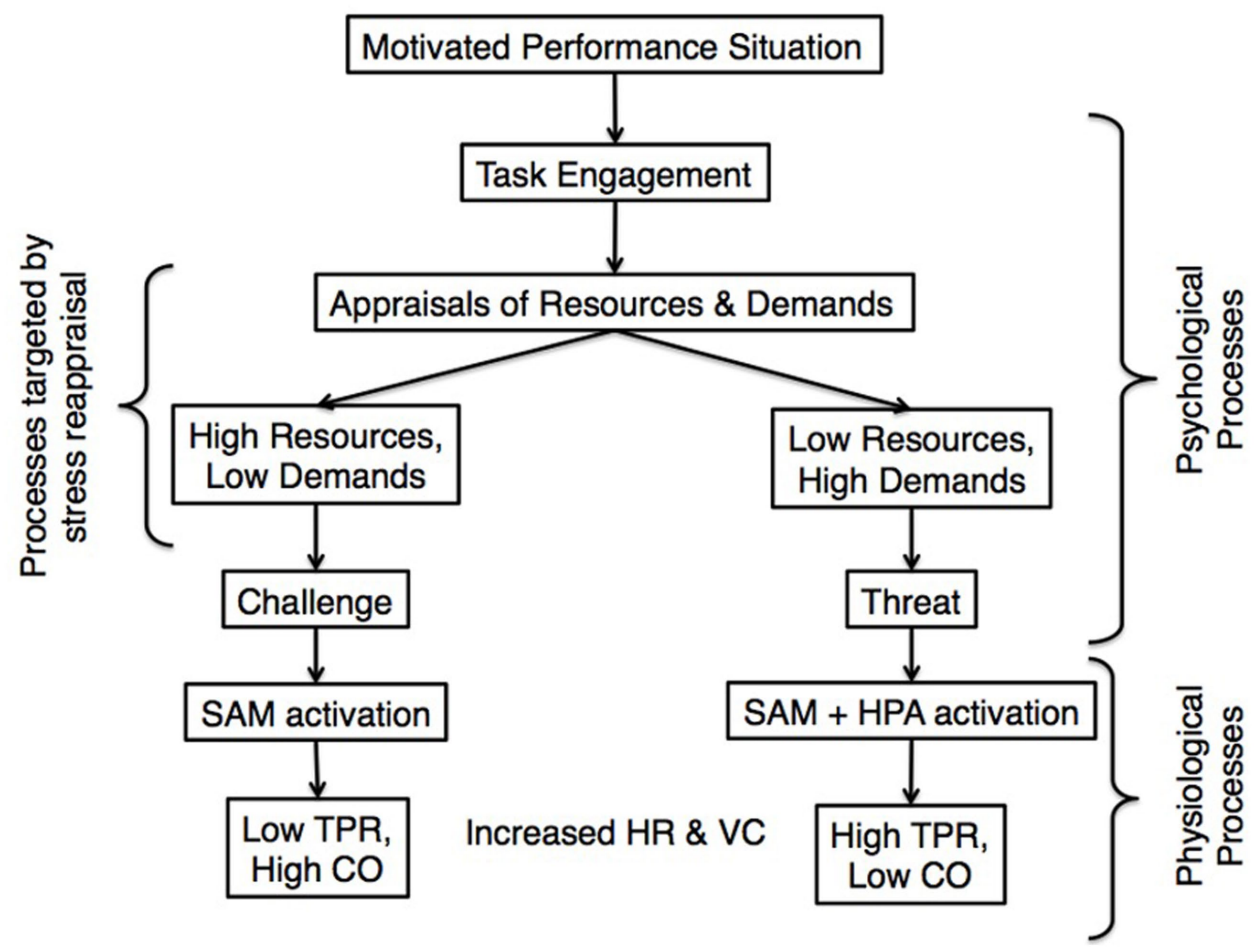
Overview of the psychological and physiological processes of the BPS model of challenge and threat. Stress reappraisal seeks to promote challenge responses by highlighting stress’s adaptive benefits. SAM, sympathetic-adrenal-medullary axis; HPA, hypothalamic–pituitary–adrenal axis; HR, heart rate; VC, ventricular contractility; TPR, total peripheral resistance; CO, cardiac output (e.g., Jamieson, 2017).

A fundamental principle of the BPS model of challenge and threat is the notion that appraisals of demands (e.g., uncertainty or effort) and resources (e.g., skills/ability) interact to cause challenge and threat-type responses in stressful situations, such as performing a difficult piece of music (see Blascovich and Mendes, 2010; Jamieson et al., 2018b; Mendes and Park, 2014 for reviews). Thus, appraisals are the fulcrum of the BPS model (see Figure 1 for a depiction of processes). The *type* of stress people experience depends on resource and demand appraisals (Jamieson, 2017). Challenge-type response occurs when appraisals of coping resources exceed appraisals of situational demands (i.e., “I believe I can handle this”), whereas threat-type response manifests when perceived demands exceed available resources (i.e., “This is too much for me”). Stress responses are not simply the result of facing a difficult situation or pressure; rather, individuals play an active role in constructing their stress responses. For example, consider two musicians in an orchestra, Alex and Jamie, who are about to perform for a large audience. Both have similar responsibilities within the orchestra and comparable training and experience. However, Alex is eager to engage with difficult challenges, while Jamie is more uncertain and anxious about the performance. When these musicians are tasked with performing a difficult piece, Alex is more likely to experience challenge, appraising the stressful situation as an opportunity to grow their skills and demonstrate competence, while Jamie is more likely to experience threat, concerned about appearing incompetent and failing. Debilitative MPA is akin to Jamie’s experience of threat in this scenario. On the other hand, facilitative MPA is more likely to follow Alex’s mindset.

While the BPS model emphasizes the active role individuals play in appraising and responding to stress, these cognitive appraisals not only shape emotional experiences but also trigger differential physiological processes, as well as motivational and behavioral outcomes. First, how an individual perceives a situation—whether as a challenge or a threat—has direct implications for their biological stress response. Specifically, these psychological response patterns interact with two primary biological stress axes: the sympathetic-adrenal-medullary (SAM) axis and the hypothalamic–pituitary–adrenal (HPA) axis. All stress responses, whether challenge or threat, initiate SAM activation, which triggers the synthesis and release of catecholamines—particularly epinephrine (i.e., adrenaline) and norepinephrine (see Figure 1). These catecholamines play a crucial role in preparing the body for action by increasing ventricular contractility and dilating blood vessels, ensuring that oxygenated blood reaches the brain and major muscle groups (Brownley et al., 1999). In challenge-type stress responses, SAM activation increases cardiac output (CO)—the amount of blood pumped through the cardiovascular system per minute—along with a decrease in vascular resistance (i.e., total peripheral resistance or TPR). Challenge responses are also marked by a rapid mobilization of resources and a quick return to homeostasis once the stressor has passed. On the other hand, threat-type stress responses also engage the HPA axis, which counteracts the anabolic effects of SAM activation. When we perceive demands as exceeding available resources, the body seeks to concentrate blood in the core of the body (i.e., increased TPR) and produces catabolic hormones (e.g., cortisol, the end-product of HPA activation) in anticipation of harm or social defeat.

Challenge-and threat-type stress responses also exhibit differential motivational and behavioral outcomes. Specifically, challenge-type stress states are linked to approach motivation, while threat responses are associated with avoidance motivation (e.g., Jamieson and Mendes, 2016; Jamieson et al., 2013). Motivational orientation is vital for performing music; an approach orientation could result in trying to perform a piece well, whereas an avoidance orientation could result in trying not to make mistakes. Across various performance settings, approach motivation is more beneficial for performance relative to avoidance (see Elliot, 2013 for a review). These motivational shifts also have important implications for cognitive and behavioral performance, particularly in the context of music performance. Research shows that challenge states tend to be linked to enhanced cognitive and physical performance compared to states of relaxation or non-stress (e.g., Blascovich et al., 1999; Brooks, 2014; Dienstbier, 1989; Jamieson et al., 2010). In contrast, threat responses impair short-term cognitive performance and promote cognitive rigidity, as well as negative health such as accelerated “brain aging,” and cardiovascular disease risk (e.g., Jefferson et al., 2010; Matthews et al., 1997).

In sum, the BPS model of challenge and threat provides a valuable framework for understanding how stress responses manifest in complex, high-pressure situations. Given that performing in front of an audience is inherently stressful, musicians navigate evaluative stressors by assessing (usually implicitly) whether their available resources are sufficient to meet the challenges they face. While threat-induced stress responses can impede learning, retention, and performance, challenge responses, which are more approach-oriented, can facilitate learning and performance and even contribute to health protection (Jamieson et al., 2018a).

### 2.2 Classic theories

**Figure 2:**
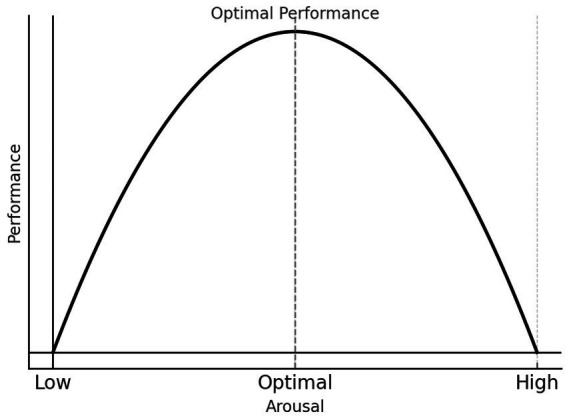
The Yerkes-Dodson “Law.” Source: modified from Özel (2024), p. 17.

While conceptualizing MPA within the framework of BPS models provides a multifaceted understanding of stress in MPA contexts and offers potential for mitigating the negative effects of MPA on musicians’ health and performance, discussions of such models are relatively rare in the MPA literature. In contrast, a more common model used to explain the conditions that contribute to “helpful” vs. “harmful” anxiety in MPA is what is sometimes referred to as the Yerkes-Dodson “law,” shown as an inverted U model of optimal performance (Yerkes and Dodson, 1908; Kirchner et al., 2008; Steptoe and Fidler, 1987; Sinico da Cunha and Winter, 2013; Wilson and Roland, 2002; see Figure 2).

The inverted U model emphasizes the importance of identifying an optimal *level* of stress—not type as in the BPS model—arguing that performance is most effective when stress levels are moderate. Based on foundational experiments by Yerkes and Dodson from 1908 and subsequent studies derived from this classic work, the inverted U model posits that both low and high stress levels impair performance, while moderate stress facilitates it. This line of research often focuses on strategies that help performers achieve and maintain an optimal level of arousal, either by upregulating low stress states or downregulating high stress states (Steptoe and Fidler, 1987; Wilson and Roland, 2002).

However, there are key limitations to the assumptions of the inverted U model that necessitate a reconceptualization of MPA along BPS lines. One major issue is its unidimensional approach to stress arousal (or sympathetic nervous system activation), treating stress as a singular concept without distinguishing between the different types of stress responses. That is, the core question for understanding MPA processes and outcomes should not be simply “How much stress?” but rather “What type of stress?” As Hanoch and Vitouch (2004) highlight, this narrow view of arousal—framing it as a continuous scale from low to high stress—fails to account for the complexity of stress states. However, this perspective has had an oversized influence on the MPA literature, leading to an oversimplification where arousal levels are often equated directly with anxiety or general stress without considering how distinct psychological and physiological responses to stress can shape performance outcomes differently.

**Figure 3:**
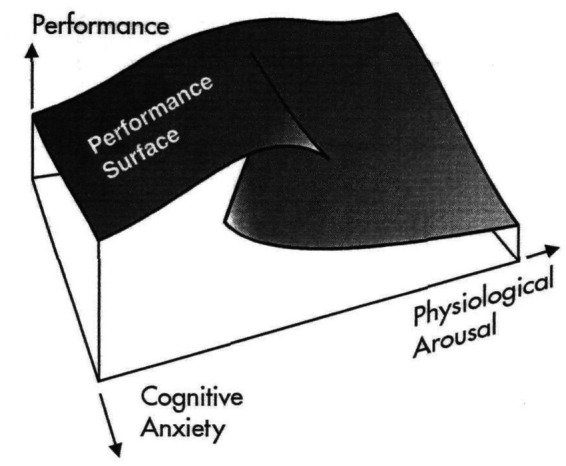
The cusp catastrophe model of anxiety and performance. Reprinted with permission, from Hardy (1996).

A more nuanced approach that attempted to differentiate the physical or somatic aspects of anxiety and the cognitive components was subsequently introduced after the earlier inverted U models. In this regard, Valentine (2004) references Fazey and Hardy’s Cusp Catastrophe Model of Anxiety and Performance, which was originally developed in the context of athletic performance. This model presents a more dynamic view of anxiety’s effects on performance, suggesting that when cognitive anxiety remains low, the inverted U curve still holds, even when physical arousal is elevated. However, when both cognitive anxiety and physical arousal reach high levels, the relationship between anxiety and performance can take a dramatic turn, with performance quality deteriorating catastrophically (see Figure 3).

This model reconceptualizes the basic inverted U-shaped curve by showing a sudden and drastic loss in performance quality as high anxiety levels are reached, rather than the gently sloping downward curve shown in Figure 2. However, like the inverted U framework, the Cusp Catastrophe Model fails to account for individual-level stress appraisals’ multifaceted and nuanced effects. Instead, it attributes anxiety primarily to situational stressors—such as personal resources or task difficulty—while minimizing the performer’s personal experience of these stressors.

From a BPS model perspective, debilitating performance anxiety arises from a threat-based pattern of appraisals, where the individual perceives the demands as exceeding their available resources. This reframing suggests that the key to effective MPA self-regulation is not about keeping anxiety or arousal levels moderate, nor simply “dampening stress.” Instead, it lies in shifting the ratio of demand and resource appraisals, enabling performers to adopt more functional stress responses in performance settings. Therefore, a potential “catastrophic” performance scenario can be reframed as a challenge or “excitement” situation, where cognitive and bodily resources rise to meet the demands at hand.

Additionally, it is essential to recognize that both challenge and threat are high-arousal states, and that the arousal aspect alone from the Yerkes-Dodson “law” does not determine anxiety levels and how performance is affected. Research based on the BPS model reveals that regulating between high arousal states—such as transitioning from threat to challenge—is not only possible but also beneficial to performance. Brooks (2014) demonstrated that shifting from one high-arousal state (threat) to another (challenge/excitement) is more effective than attempting to regulate from a high-arousal state to a lower one (calm).

**Figure 4:**
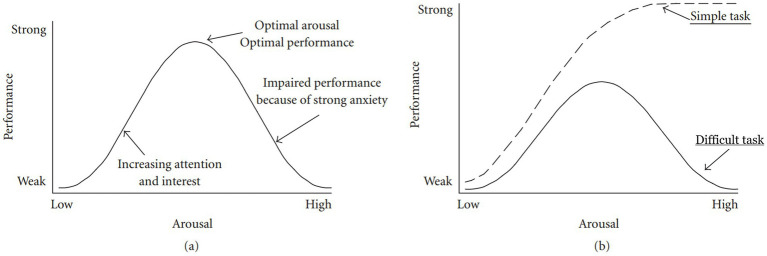
“Hebbian” (graph a) vs. original (graph b) version of Yerkes-Dodson findings. Source: modified from Diamond et al. (2007, p. 3).

Another key limitation of the basic inverted U-shaped model is its misrepresentation of the original Yerkes-Dodson findings, which emphasized the crucial role of task difficulty in the relationship between arousal and performance. The original experiments of Yerkes and Dodson showed that high levels of arousal did not negatively affect performance on easy tasks; rather, it was only when tasks became more difficult that high arousal was shown to be debilitating (Yerkes and Dodson, 1908). Unfortunately, influential studies on emotion and learning in the 1950s oversimplified or misrepresented the complexity of Yerkes and Dodson’s findings, often reducing their law to a simple inverted U-shaped curve. To address this distortion, Diamond et al. (2007) compare what they call the “Hebbian version” of the Yerkes-Dodson law—the widely accepted interpretation for the last 50 years—and the original Yerkes-Dodson findings. This side-by-side comparison, presented in Figure 4, highlights the importance of factoring in task difficulty alongside arousal levels to more accurately understand how these variables interact and affect performance.

The Hebbian version (Figure 4a) presents only the inverted U-shaped curve, while the original version (Figure 4b) illustrates that task difficulty influences how stress arousal affects performance. The performance of simple or well-learned tasks does not suffer from high arousal, but the performance of complex or uncertain tasks does. Contrary to common interpretations in much MPA literature, Yerkes and Dodson concluded that task difficulty, not merely arousal level, is the key determinant of whether a stressful situation leads to facilitative or debilitative arousal. In fact, when undergoing tasks with a low level of difficulty, arousal is rather positively correlated to performance. As Diamond et al. (2007) succinctly state, the issue with using ‘task difficulty’ as a critical factor in understanding arousal-performance interactions is that it is subjective. Task difficulty is determined by the performer’s subjective evaluation or appraisal of the task, rather than the task itself.

In light of this, emotion regulation in MPA should shift focus from simply lowering arousal levels, as suggested by the often misinterpreted Yerkes-Dodson “law,” to modifying appraisals when arousal is high. This approach ensures that the performer perceives their resources as adequate to meet the demands of the task. This perspective aligns with BPS models, where maladaptive MPA arises when demands are seen as exceeding available resources (i.e., when the individual feels threatened), whereas facilitative MPA occurs when resources are perceived as exceeding demands. Given this understanding, the next crucial step is to explore how individuals can use the BPS model framework to mitigate the impact of MPA and enhance performance.

## 3 Regulating stress in MPA contexts

### 3.1 Stress optimization

Contrasting with traditional relaxation or downregulation techniques that aim to reduce stress or remove stressors, recent advances in affective science promote a “stress optimization” perspective. This perspective encourages engagement with rigorous but useful stressors—like musical performances—while fostering challenge-type stress responses (Crum et al., 2023; Jamieson et al., 2018a,b; Journault et al., 2024). It does so by changing the individual’s meaning of stress, stressors, and how physiological stress responses impact performance. Stress reappraisal is a technique developed from the BPS model that helps individuals interpret physiological arousal (e.g., racing heart, sweaty palms) as signs of engagement and readiness rather than anxiety (Jamieson et al., 2010). Thus, it helps individuals reframe their stress responses as functional and performance-enhancing (Jamieson et al., 2012; Jamieson and Hangen, 2022; Oveis et al., 2020). For instance, in a double-blind field experiment, community college students who received stress reappraisal instructions performed better on exams, reported lower anxiety, and demonstrated higher resource appraisals than those who received placebo instructions (Jamieson and Hangen, 2022; Jamieson and Mendes, 2016). Similarly, laboratory studies have shown that when people appraise stress as functional rather than debilitating, they exhibit healthier neuroendocrine and cardiovascular responses and less observable anxiety in high-pressure tasks (Beltzer et al., 2014; Jamieson et al., 2012).

Stress reappraisal may be useful for musicians to help promote challenge-type responses during performances, but appraisal processes fluctuate across contexts, even for the same individual. To illustrate, an expert skier may feel confident tackling a difficult trail but appraise a new musical piece in a class setting as beyond their ability. Because appraisal-based interventions are context-dependent, broader cognitive frameworks are needed to enhance their effectiveness across different learning and performance situations. Transferring reappraisal messages can also be difficult for people if they encounter initial difficulties. For example, consider a musician seeking to reappraise their stress as functional before a performance. The musician may try to appraise their stress as a resource, but if they subsequently receive poor reviews or evaluations, they may conclude that reappraisal is not useful and will not apply the message to other domains in the future.

**Figure 5:**
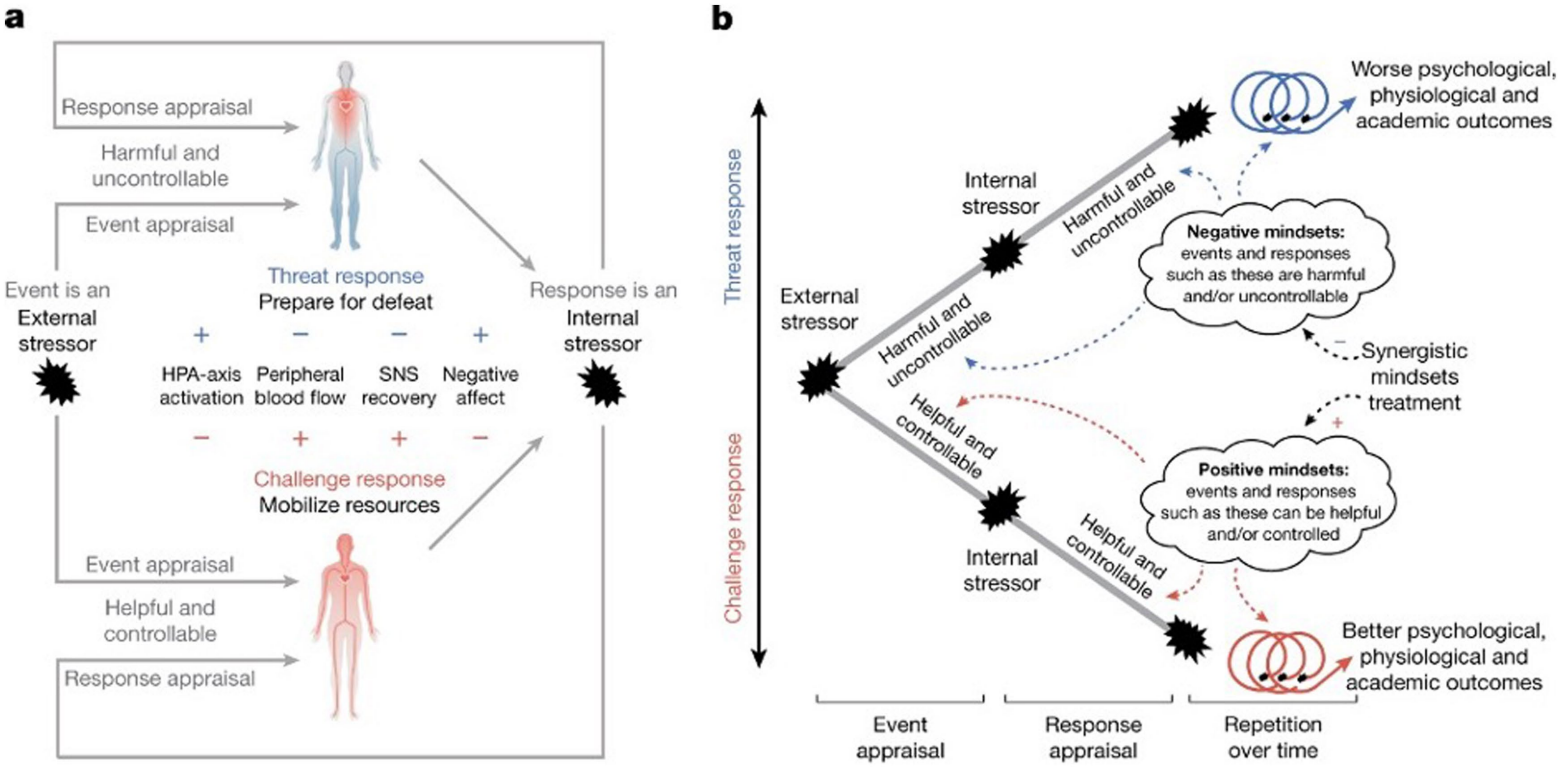
Individuals appraise both acute stressful events and their stress responses **(a)**; and their mindsets shape appraisals and responses **(b)**. Stressful events, such as a challenging learning exercise or an argument with a friend, are appraised as either harmful and uncontrollable or more helpful and controllable, cultivating threat or challenge response tendencies, respectively. Then, the meaning of stress and the stress response is appraised as either distressing and non-functional (harmful and uncontrollable) or as a resource that helps one address situational demands (helpful and controllable), which results in further threat-or challenge-type stress responses, respectively. Individuals who respond with an optimized challenge-type stress response engage with and respond to future stressors more adaptively in a self-reinforcing, positive recursive feedback cycle that results in improved outcomes (from Yeager et al., 2022).

To solve the transfer problem across contexts and in the face of failures, a promising theoretical advance integrating BPS-based stress reappraisal with mindset theory is “synergistic mindsets” (Yeager et al., 2022, see Figure 5). This approach combines (1) growth mindsets—the belief that ability develops through effort, strategy, and support (Yeager et al., 2019)—with (2) stress-can-be-enhancing mindsets, which frame stress as beneficial for performance (Crum et al., 2013; Jamieson et al., 2010). These complementary beliefs provide a holistic framework for helping musicians engage with stress adaptively. This integrated approach not only helps people internalize reappraisal messaging across contexts but leveraging mindsets also discourages individuals from overgeneralizing one negative experience in a particular performance context to all future performance contexts.

The synergistic mindsets approach may be particularly effective for improving outcomes in MPA contexts. A musician with an event-focused growth mindset may believe that struggling with difficult material leads to improvement. If they also hold a stress-is-debilitating mindset, they might avoid high-pressure performances due to fear of failure. Conversely, a musician who sees stress as beneficial but believes that ability is fixed may disengage when they encounter difficulty. By simultaneously fostering both mindsets, the synergistic mindsets approach encourages musicians to view both their challenges and their stress as assets in achieving valued goals (Yeager et al., 2022).

A key advantage of synergistic mindsets is their durability. Unlike context-specific appraisals, mindsets function as cognitive “lenses” that shape how individuals appraise future situations, creating self-reinforcing cycles of adaptive stress responses. For musicians, successfully applying these mindsets in one performance can lead to broader shifts in stress perception, fostering resilience and long-term improvement. Similar to how negative feedback loops can exacerbate anxiety and avoidance, positive feedback loops can promote emotional growth and achievement (Gross, 2015). Additionally, reappraising failures as feedback can lead to lasting shifts in cognition. Research suggests that repeated engagement with strategies that reconstrue the meaning of failures can alter mental models over time, leading to more adaptive interpretations of future stressors (Uusberg et al., 2019). For musicians struggling with MPA, interventions that target synergistic mindsets have the potential to transform performance-related stress from a debilitating obstacle into a tool for growth and mastery.

While reappraising demands as resources and mindset-based strategies can help musicians engage with stress more adaptively, they are not always applicable or effective in every situation. Reappraisal-and mindset-based approaches are not “silver bullets” that work in every context for every individual. It is important to understand when mitigating or downregulating stress might be helpful in MPA contexts. In contrast with stress optimization, stress mitigation is generally aimed at removing stressors or reducing the demands from the stressors. The following section briefly describes some of the more traditional stress mitigation strategies used by musicians when stress optimization is not applicable.

### 3.2 Stress mitigation

Musical skill acquisition, practice, and repetition create prepotent responses (i.e., playing music becomes proceduralized with sufficient training/practice). Stress potentiates these prepotent responses, especially under conditions of social evaluation such as playing a concert (e.g., Jamieson and Harkins, 2010). That is, under stressful conditions, people default to executing cognitive and behavioral response patterns that are well-learned/well-trained, and the stress response helps facilitate these types of responses (e.g., Beilock and Carr, 2001). As reviewed in the BPS selections above, as long as people have the skills/training to perform at a high level (i.e., resources are high), approach oriented challenge responses are beneficial. However, if trained musicians begin to doubt their skills or perceive the performance context as overwhelming, this breaks down proceduralization. The musician focuses on “not making mistakes” and performing each component of their skill.

For instance, consider a violinist performing in front of an audience. The violinist can appraise their resources (e.g., skills, ability, training) as exceeding demands, optimize their stress response and do well as the stress potentiates their proceduralized actions. Alternatively, suppose the violinist appraises demands as overwhelming (e.g., the performance has direct consequences for their career) rather than “leaning into” their stress. In that case, they worry about their finger placements and intonation. Because breaking a proceduralized act (i.e., playing the violin) into component parts (e.g., finger placement, bow movement) does not allow stress to potentiate those well-learned prepotent responses, the violist’s timing is off, and they underperform. Thus, in MPA contexts, stress optimization regulation approaches aim to support trained musicians in maximizing outcomes. What about novices or new learners, though? When individuals objectively do not (or could not) possess the resources to address situational demands, no reappraisal or mindset will help support performance. This would be akin to taking a novice violinist, placing them in a philharmonic orchestra, and asking them to perform in a concert. In cases where optimization is not viable, especially for novice or beginner musicians in the context of MPA, mitigating stress and downregulating threat can be useful tools. Below, we review a limited list of mitigation approaches, including behavioral, cognitive and chemical approaches.

Some behaviors can be adopted by musicians to mitigate stress levels, such as controlled breathing exercises, practice performances and visualizations. Controlled breathing exercises can be an effective strategy for managing overwhelming, maladaptive MPA (Mac Afee and Comeau, 2020). These strategies can be particularly helpful for novices as they prepare for early evaluations of their musical ability. For instance, deep breathing exercises that use shorter inhalations and longer, slower exhalations (also called sighing) can increase positive affect and reduce physiological arousal (i.e., downregulate threat) relative to other types of controlled breathing, such as box breathing (equal inhale, hold, and exhale ratios) and hyperventilation with retention (Balban et al., 2023). In addition, nasal breathing triggers synchronized electrical activity in brain regions implicated in affective responses (Balban et al., 2023). Incorporating nasal breathing and long, slow exhalations into practice can help quickly calm an overly anxious musician. Such an approach also has the potential to shape future appraisals if the performance goes well—that is, individuals may appraise evaluative performances as less demanding if they are successful at downregulating their threat responses.

Frequent practice performances or visualizations can also help downregulate threat by boosting resource appraisals via providing skills or increasing familiarity; these can help performances seem more manageable (Wilson and Roland, 2002; Huang and Yu, 2022). To be most effective, practice performances should follow a graded approach, starting with informal settings—such as playing for close friends and family in a relaxed environment—before gradually increasing audience size and formality. This progressive exposure helps musicians become accustomed to their typical physiological and psychological responses to stress, making these reactions feel more predictable and less alarming. Similarly, mentally simulating a performance allows musicians to familiarize themselves with the concert setting, reducing novelty and uncertainty (Roland, 1994, p. 28). Many conservatories now incorporate virtual reality (VR) training, where musicians practice performing in simulated high-pressure environments projected on large screens (Zhukov, 2019). Research suggests that VR-based exposure effectively reduces debilitating MPA and enhances performance outcomes (Orman, 2004; Bissonnette et al., 2016).

Other approaches to reducing debilitating MPA, such as anxiety defusion, self-distancing and anxiety acceptance, are more cognitive. Anxiety defusion involves creating psychological distance from the emotional experience by labeling and defining negative emotions, such as threat in MPA. By distinguishing the anxiety itself from the ability to think about it, individuals can gain some control over what would otherwise be an undefined emotional experience (Ellsworth, 2013). Likewise self-distancing (i.e., considering oneself in the third person) and emotion differentiation techniques such as clearly labeling affective responses can reduce catastrophization tendencies and allow individuals to select successful active regulation strategies (e.g., Barrett et al., 2001; Kross and Ayduk, 2017). Observing anxiety rather than becoming consumed by it can help individuals identify resources and recognize that the emotional response is separate from the self, which in turn reduces the feeling of being overwhelmed. Anxiety acceptance goes a little further down this route by encouraging individuals to embrace anxiety rather than resist it. Resistance, often seen in attempts to suppress or avoid, tends to amplify negative experiences, especially in social settings (Blackledge and Hayes, 2001; Peters et al., 2014). Acceptance, on the other hand, involves recognizing anxiety as a temporary, normal emotion that can be tolerated without the need for suppression. This approach reduces the perceived intensity of anxiety and the sense of uncontrollability, which often magnifies anxiety symptoms and produces a cycle of panic (Kashdan and Rottenberg, 2010). Acceptance strategies can help musicians acknowledge anxiety as part of the performance experience, reducing the tendency to experience threat.

Finally, some mitigation approaches can be chemical. To regulate high stress levels in high pressure settings and in the absence of successful alternative strategies, some musicians use beta-blockers to ensure on-stage performance. Beta-blockers are used to attenuate physical symptoms of MPA, such as racing heart, hand tremors, or sweaty palms (Kenny, 2011). However, these pharmaceutical interventions only alter biological processes and do not regulate psychological processes. Musicians unaware of this distinction may develop psychological dependence, relying on medication to feel in control. While performers on beta-blockers may find it easier to appraise performance situations as manageable because physical symptoms of anxiety are dampened, there is a potential to lose the benefits of stress in that the music may sound “flat” and “unemotional” if the physiological stress responses are blocked.

## 4 Discussion and conclusion

MPA is a complex phenomenon that has the potential to both hinder and enhance performance, depending on how it is appraised and regulated. Traditional research has largely focused on the debilitating effects of MPA. However, emerging perspectives emphasize the potential for optimizing stress responses by adopting adaptive mindsets and regulation strategies to transform anxiety into a performance asset. Because MPA is a stress state, it is similar to other forms of anxiety and other high arousal affective states like excitement or anger. Starting from the perspective that MPA is a form of stress then allows stress regulation tools to be applied to MPA contexts. Unfortunately, lay theories of stress suggest stress is always bad and should be avoided to help support health, performance, and wellbeing. Stress is equated with distress. Unsurprisingly, when people were asked what the best advice would be for performing under stress, over 90% indicated that remaining calm and relaxed was best (Brooks, 2014). Thus, it is unsurprising that much work on stress regulation focused on mitigation and attenuation. Stress was seen as a problem to be solved rather than an opportunity to be harnessed.

The BPS model of challenge and threat is built on classic theories of emotion and stress, but emphasizes the multifaceted nature of stress and the potential facilitative effects of approach-oriented stress states like challenge. This model introduced the idea that improving outcomes under stress is not about reducing the *amount* of stress but changing the *type* of stress. As reviewed here, cognitive appraisal processes are at the core of the BPS model and operate to determine physiological responses to stress. When resources exceed perceived demands, facilitative challenge responses follow, with less functional threat responses following when resources are insufficient to meet demands. Thus, shifting appraisals using psychological interventions can be powerful tools for promoting more adaptive stress responses in music performance settings. Recent advances from the BPS model have integrated mindset processes which are broad and situation-general lenses through which people interpret the world. Building interventions that incorporate stress reappraisal ideas (i.e., presenting the stress response itself as a resource because it mobilizes resources), with general mindsets that stress-can-be-enhancing and growth mindsets (i.e., that one’s skills can be developed with persistence and good strategies) can yield powerful effects (Yeager et al., 2022). While this “synergistic mindset” approach has yet to be tested in MPA contexts, there is much potential for this optimization approach to improve musicians’ performances and wellbeing.

Musicians experience MPA because they are invested in and care about their performance. If musicians did not care or had no motivation to perform, there would be no stress. There also would not be very many opportunities for a disengaged musician. So, like high level athletes, expert musicians need to be invested and “amped up” to do well in performances. Thus, MPA is normal for musicians to experience and helping them utilize their stress/anxiety in a most helpful way should be a major focus of regulation research. Existing regulatory approaches, though, have more strongly focused on mitigating the negative effects of stress rather than optimizing it. While those approaches can be helpful for musicians just starting out or still in the early stages of learning, or if a musician has accompanying mental health difficulties that make stress optimization unfeasible, promoting facilitative challenge responses in the MPA context can help musicians reap the largest regulatory benefits.

In addition to possible performance benefits which may result from promoting stress optimization in musicians, there exists potential for health benefits as well. Notably, the profile of cardiovascular and neuroendocrine responses that are characteristic of threat-type stress responses (increased TPR, elevated cortisol, and a slower return to homeostasis after stress offset) has been shown to elevate risks for cardiovascular disease (e.g., Gordon and Mendes, 2021; Jamieson et al., 2012; Berry Mendes et al., 2007). Future research in music science could explore the potential health impacts of MPA from a BPS perspective.

The synergistic mindset approach reviewed here is also scalable. Psychological interventions that deliver content via online methods allow those interventions to be administered to a large sample of musicians at a low cost. Early delivery at scale could even help buffer musicians from debilitative MPA. That is, the synergistic mindset intervention is best conceptualized as a preventive tool that can stop experiences of anxiety early on in skill acquisition from “snowballing” via negative recursive processes into debilitating performance anxiety.

While stress plays an important role in musicians’ functioning and performance outcomes, and regulating stress is vital to optimizing outcomes, research cannot simply administer interventions and “set it and forget it.” Rather, it is necessary and important to build studies that seek to understand heterogeneity and assess generalizability in meaningful ways (Bryan et al., 2021). Not every musician should be expected to respond to or benefit from stress optimization the same. We touched on one potential variable – expertise level – that could play a role in how regulation should be applied. Still, various other potential psychological or demographic moderators could emerge. For instance, musicians playing stringed instruments may respond differently than vocalists to different regulatory approaches. Or, stress may facilitate performance differentially in musicians who are formally trained vs. those who are self-taught. Understanding the “whens and whys” of how stress impacts performance and how interventions impact musicians is an important endeavor for music science. As this research domain builds out, researchers must keep in mind testing for sources of heterogeneity and probing questions of generalizability.

In conclusion, this paper reviewed some theoretical models of stress and their relevance to MPA, highlighting how musicians can cultivate more adaptive responses to evaluative pressure. Rather than viewing MPA solely as a problem to be mitigated, a more nuanced approach considers how musicians can develop psychological flexibility, employing diverse strategies to regulate stress based on situational demands. Finally, we suggest that lay conceptualizations and discussions of stress advance a flawed narrative about the potential for people to address difficult challenges. The predominant societal response to increasing levels of anxiety and stress–across music to education to vocational domains, to name a few–has argued that we should *expect less* of people. In other words, removing stressors has been thought to be the best way to alleviate stress-related problems. However, people can do hard things. Scientists can thus focus on supporting musicians experiencing MPA to achieve. It is critical that people face normative stressors in their lives to grow, learn, and thrive. Nobody innovates by staying within their comfort zones. The science of stress suggests musicians must face difficulties and challenges as they develop their skills and abilities. Science and art are intertwined; applying advances in stress science can help support musicians in creating inspiring art.
