# Correction: A review of music performance anxiety in the lens of stress models

**DOI:** 10.3389/fpsyg.2025.1707765

**Published:** 2025-10-21

**Authors:** A. J. Twitchell, A-A. Journault, K. K. Singh, J. P. Jamieson

**Affiliations:** ^1^Independent Researcher, Florianopolis, Brazil; ^2^Department of Psychology, University of Rochester, Rochester, NY, United States

**Keywords:** music performance anxiety, biopsychosocial model, stress, stress optimization, review

Kenny (2009) was not cited in the article. The citation has now been inserted in the section Introduction, Paragraph 2 and should read:

“Although definitional discrepancies exist, Kenny (2009) definition has become increasingly relied upon describing MPA as an “experience of marked and persistent anxious apprehension related to musical performance” (Kenny, 2009, p. 433), characterized by a combination of affective, cognitive, somatic, and behavioral symptoms. It may harm the quality of the performance but does not necessarily do so.”

The reference for Kenny (2009) was not included in the reference list. The reference to include should be:

Kenny, D.T. (2009). “Negative emotions in music making: performance anxiety,” in *Handbook of Music and Emotion: Theory, Research, Applications*, eds. P. Juslin, and J. Sloboda (Oxford: Oxford University Press), 425–451.

The original version of this article has been updated.

